# Detection and counting of pigment glands in cotton leaves using improved U-Net

**DOI:** 10.3389/fpls.2022.1075051

**Published:** 2023-01-09

**Authors:** Lixuan She, Nan Wang, Yaxuan Xu, Guoning Wang, Limin Shao

**Affiliations:** ^1^ State Key Laboratory of North China Crop Improvement and Regulation, College of Mechanical and Electrical Engineering, Hebei Agricultural University, Baoding, China; ^2^ State Key Laboratory of North China Crop Improvement and Regulation, College of Agronomy, Hebei Agricultural University, Baoding, China

**Keywords:** cotton leaf, pigment gland, gossypol, U-Net, small objects, semantic segmentation

## Abstract

Gossypol, as an important oil and raw material for feed, is mainly produced by cotton pigment gland, and has a wide range of applications in the fields of pharmaceutics, agriculture and industry. Accurate knowledge of the distribution of pigment gland in cotton leaves is important for estimating gossypol content. However, pigment glands are extremely small and densely distributed, manual counting is laborious and time-consuming, and difficult to count quickly and accurately. It is thus necessary to design a fast and accurate gland counting method. In this paper, the machine vision imaging technology is used to establish an image acquisition platform to obtain cotton leaf images, and a network structure is proposed based on deep learning, named as Interpolation-pooling net, to segment the pigment glands in the cotton leaf images. The network adopts the structure of first interpolation and then pooling, which is more conducive to the extraction of pigment gland features. The accuracy of segmentation of the model in cotton leaf image set is 96.7%, and the mIoU (Mean Intersection over Union), Recall, Precision and F1-score is 0.8181, 0.8004, 0.8004 and 0.8004 respectively. In addition, the number of pigment glands in cotton leaves of three different densities was measured. Compared with manual measurements, the square of the correlation coefficient (*R* ^2^) of the three density pigment glands reached 0.966, 0.942 and 0.91, respectively. The results show that the proposed semantic segmentation network based on deep learning has good performance in the detection and counting of cotton pigment glands, and has important value for evaluating the gossypol content of different cotton varieties. Compared with the traditional chemical reagent determination method, this method is safer and more economical.

## Introduction

Cotton is an important economic crop. It is not only the main source of high-quality natural fibers, but also the resource of protein and oil (Qu et al., 2017). Cotton pigment gland is a widely distributed unique tissue structure of cotton plants in most organs of cotton plants except pollen and seed coat. It is the dark brown opaque spots in cotton leaves (Liu et al., 2010; Zhang et al., 2021). Cotton pigment glands contain gossypol and other terpenoid aldehydes (Gao et al., 2020). Gossypol is a yellowish-brown polyphenol pigment insoluble in water but soluble in organic solvents, which is synthesized in the roots and carried in various organs of cotton plants (Zhao et al., 2020). Gossypol is widely used in pharmaceutical, agricultural and industrial application. For agricultural application, gossypol (Kong et al., 2010; Krempl et al., 2016) is used to control crop diseases and insect pests due to its good insect resistant characteristic and also for the control of rodent damage in the field due to its fertility resistance (Hahn et al., 1981). In pharmaceutics, the antifertile trait of gossypol is made use of in the manufacturing of contraceptives (Qian and Wang, 1984), drugs that inhibit the growth and proliferation of tumor cells (Badawy et al., 2007; Ni et al., 2013), and it has obvious effect on the treatment of gynecological diseases (Hsieh et al., 2020). However, the toxicity of gossypol limits its wide application as an important oil and feed raw material. Excessive intake of gossypol will cause human and animal digestive dysfunction and gastric mucosa damage (Lordelo et al., 2005; Gadelha et al., 2014). Therefore, the detection of gossypol content in cotton plants is very crucial in the improvement of the economic value of cotton.

At present, chemical reagent method is most commonly used for the detection of gossypol content such as High Performance Liquid Chromatography (HPLC) (Benbouza et al., 2002), Capillary Electrophoresis (Liu et al., 2005) and Ultraviolet Spectrophotometry (Przybylski et al., 2006). However, they will not only damage the samples, but also are costly. The relationship between cotton pigment glands and gossypol is close and complex. Pigment glands are the storage organ for gossypol and derivatives of gossypol. Their distribution density is positively correlated with the glanded cotton (Wilson and Smith, 1976). The number of pigment glands is an important predictor for estimating the gossypol content in glanded cotton. Pigment glands are small with an average diameter of 100 ~ 400 μm (Qian et al., 2017) and densely distributed, and the accurate counting of pigment glands is difficult to perform manually. At present, there are some methods for counting the pigment glands of cotton. The mainstream method used is the microscope observation method. Wang et al. (2018) used stereomicroscope to take pictures of the leaves of four species of cotton plants, *Gossypium barbadense L.*, *Gossypium hirsutum L.*, *Gossypium herbaceum L.*, and *Gossypium arboretum L.*, and counted the number of pigment glands in total, then divide it into the total area and got the number per square centimeter. This process is time-consuming and laborious, and the experience of labors may influence the result, and thus the accuracy of the count number can’t be insured.

Computer vision or more precise plant phenotyping has been proved to be a reliable too in the field of plant biology, and the application of computer vision technology on the study of plant phenotype has achieved some fruitful results (Dee and French, 2015). The cotton pigment glands only account for a very small percentage in the whole cotton leaf image and so the small object detection method has to be used to detect the number of cotton pigment glands (Lin et al., 2014; Zhu et al., 2016). The coverage area that small objects in the image is generally dozens of pixels, or even a few pixels, with less information on the features and lacking feature expression ability. The small object detection method has always been the focal point of studies of many researchers (Li et al., 2022). At present, small object detection methods mainly are the traditional image processing method and deep learning method. The traditional image processing method mainly rely on manual analysis of image features, extraction of image features with algorithms, and distinguishing objects by feature values (Su et al., 2020). However, the feature extraction process is complex and time-consuming, and it is difficult to achieve the same detection accuracy as the detection of large object. With the improvement of computing power of equipment, deep learning has become more and more popular for image recognition and image segmentation (Tetila et al., 2019; Su et al., 2020). Different from the traditional image processing method, deep learning extracts image features from the network framework. Convolutional Neural Network (CNN) has excellent performance in exploring more deep information of images. Sun et al. (2021) introduced the segmentation masks to remove background images based on the original SSD model, which improved the detection performance of the Tsinghua-Tencent 100K and Caltech pedestrian dataset by adding context information. In Convolutional Neural Network, the shallow feature map has a small receptive field to detect small objects, while the deep feature map has a large receptive field to detect large objects. Zhou et al. (2018) proposed a novel Scale-Transferable Detection Network (STDN) by embedding a super-resolution layer in a Dense Convolutional Network (DenseNet). Firstly, the small-scale feature map is obtained, and then the large-scale feature map is obtained by reducing the number of channels of the feature map in the super-resolution layer to explicitly explore the inter-scale consistency. However, the objects are closely adjacent to each other in the single image where the small objects have dozens or even hundreds of objects to be detected (Ming, 2021), which is easy to cause detection overlapping and false detection of small objects (Goldman et al., 2019; Pan et al., 2020), not to mention the identification and counting of dense small objects, which is more challenging.

**Figure 1:**
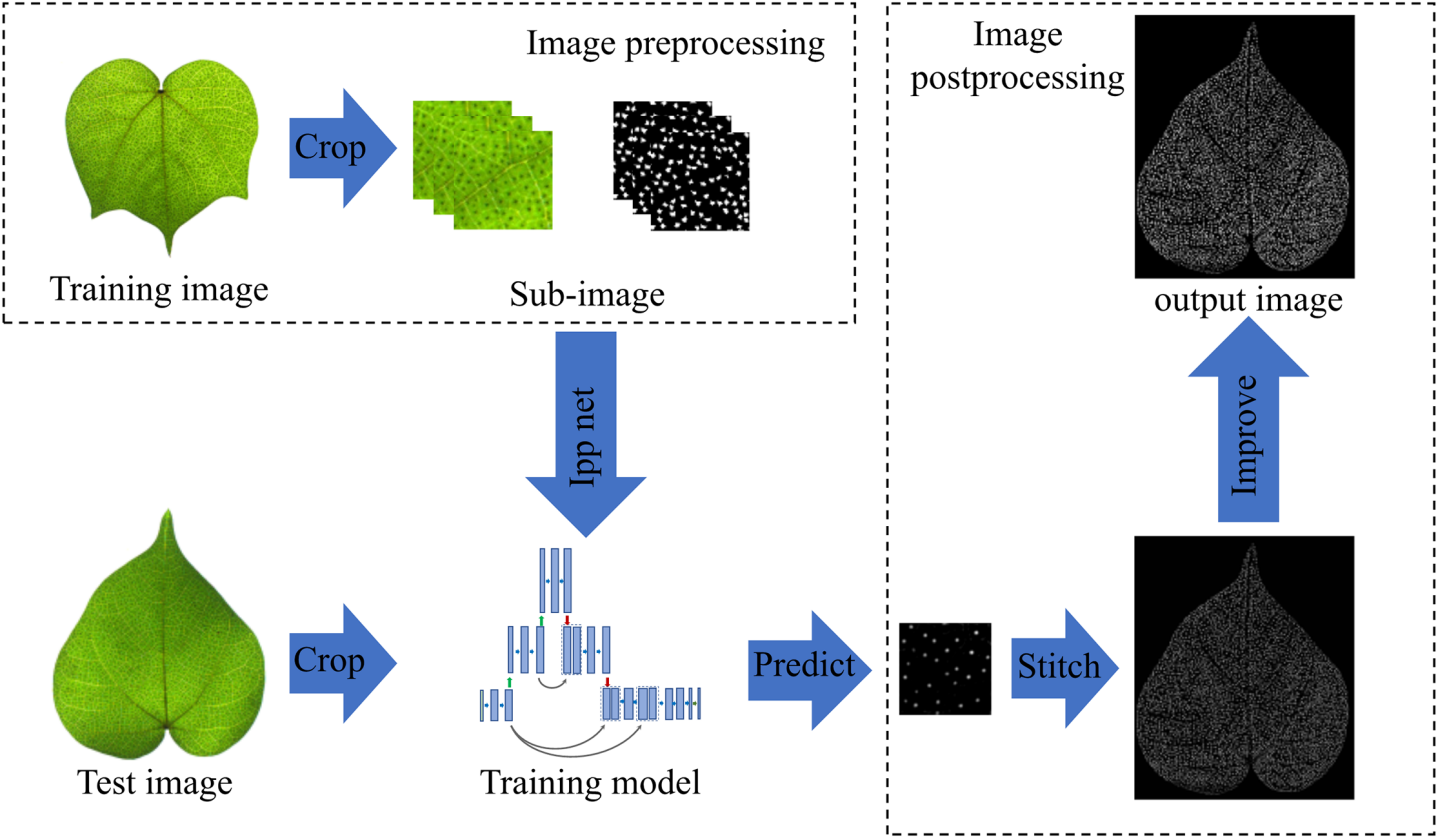
Flow chart of cotton pigment gland detection.

Pigment glands are small, numerous and densely distributed, so it is difficult to manually count them accurately. On the basis of previous research work, we inverted the structure of the classical U-Net network and designed a semantic segmentation model of pigment glands based on deep learning to realize the rapid recognition and counting of densely distributed pigment glands. The model first performs the interpolation operation for up-sampling to amplify the image features, and then performs the pooling operations to achieve multi-scale fusion of the network by fusing deep semantic information and shallow representation information. First of all, a cotton leaf image acquisition device was built to collect the RGB image of cotton leaf with a color industrial camera, and then the image was manually annotated, binary image conversion, rotation and cropping, so as to build the data set of cotton leaves. After that, the improved model was trained and validated. The shape feature filtering method was used to optimize the segmentation results to reduce the error caused by the leaf vein shadow in the image. Finally, the pigment glands in the images were counted. The flow chart is shown in **Figure 1**. In order to evaluate the segmentation performance of the network, we compared the segmentation and counting results of the improved model with original U-Net, DeepLabv3+ and manual counting. The comparison results showed that the improved model had achieved high-throughput and accurate detection of cotton pigment glands.

## Materials and methods

### Image collection

In August 2021, cotton leaf image acquisition experiments were carried out at the innovation experimental park of Hebei Agricultural University in Baoding City (38.85°N, 115.30°E), Hebei Province, China. The true leaves of seedling stage of upland cotton with a growth cycle of 35 days were taken as samples, among which those with intact leaf phenotype and uniform chlorophyll distribution were selected for cleaning and drying.

**Figure 2:**
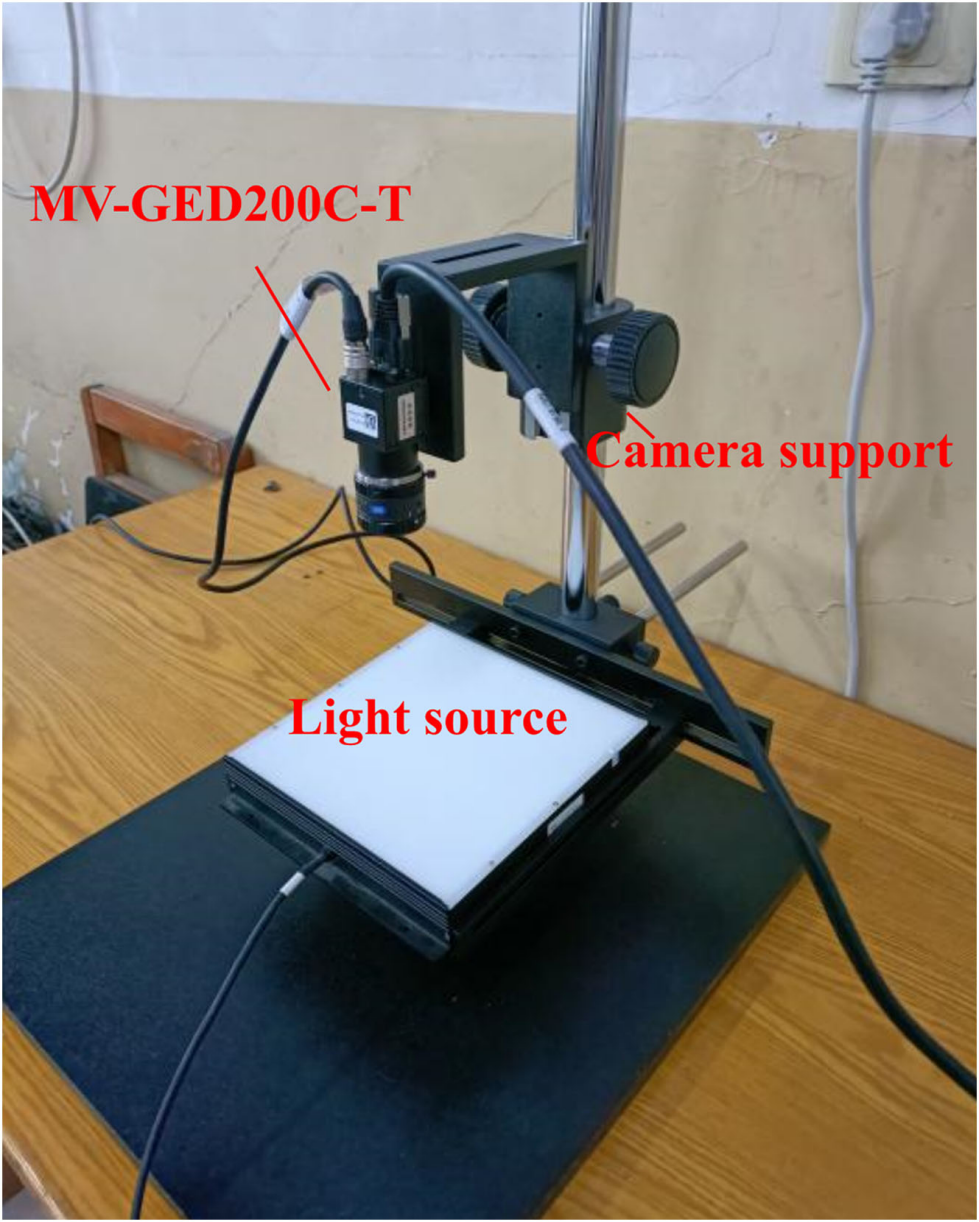
Cotton leaves image acquisition device.

An image acquisition platform was established in advance, as shown in **Figure 2**. The cotton leaf was placed under the white square light and the color mesh industrial camera was connected (MV-GED200C-T, Mind Vision, China, maximum resolution: 1600×1200) to the computer. The camera support knob was adjusted to keep the camera at an appropriate height so that the leaf can be in the camera aperture completely, and extend in as much as possible to collect cotton leaf images of different densities. The focal length and aperture of the camera lens (OPT, focal length: 12mm) were adjusted, as well as the light source controller to make the pigment glands in the cotton leaf as clear as possible. Finally, the RGB images of cotton leaves were collected by the industrial camera controlled by the industrial camera software.

### Data set processing

**Figure 3:**
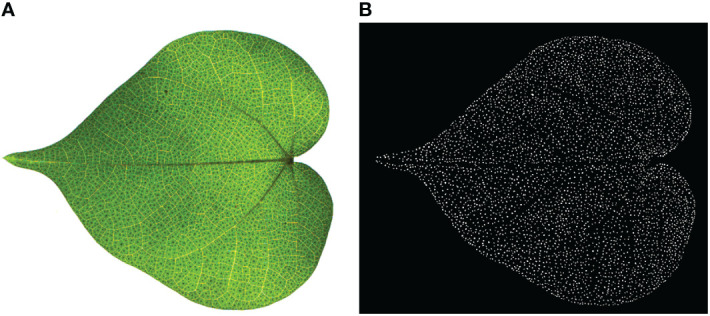
Manual labeling of cotton leaves at seedling stage. **(A)** Original image; **(B)** Annotated image. The background is in black, and the cotton pigment glands are in white.

33 RGB images of cotton leaves with a resolution of 1600×1200 were collected by the image acquisition device, and saved as BMP file. The collected 33 cotton leaf images were screened to eliminate the ones that were blurred, damaged and dark leaf images, and 9 qualified cotton leaf images were sifted out and labeled manually. The image annotation was performed by annotation tool LabelMe (Russell et al., 2008), it contained about 37,584 labeled pigment glands in 9 images, and the average labeling time of each image was about 5.5 hours. The json files generated after labeling were saved in the computer. In order to improve the generalization performance of the training model, the 9 labeled json files were converted into binary images of black and white, where the pigment glands were white and the background was black, as shown in **Figure 3**. Then, the binary images were reversed horizontally, vertically and mirrored, and 36 images were generated. Because the leaf image field of view was extensive, to allocate GPU memory was difficult. To solve the problem, the image was cropped into smaller size and 6912 sub-images of 100×100 of pixel size were generated. 80% of them were used as the training set and 20% as the validation set.

**Figure 4:**
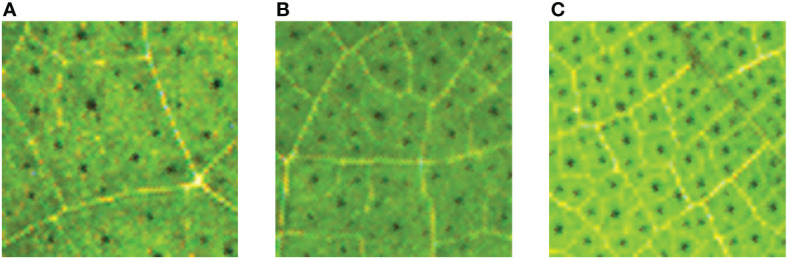
The sub-images of three datasets with different densities. **(A)** DS1; **(B)** DS2; **(C)** DS3.

In addition, we also selected another 7 clear images of different densities as the counting study of this model, and the 7 images were cropped into 453 sub-images. In order to verify the counting accuracy of pigment glands with different densities, the sub-images were divided into three grades according to the density. It is assumed that the number of pigment glands in 4.5×4.5mm area was less than 40 be level 1, the number more than 40 but less than 80 be level 2, and the number more than 80 be level 3. The three levels were categorized into three different datasets DS1, DS2 and DS3 respectively, and the number of sub-images of DS1, DS2 and DS3 was 184, 156 and 113 respectively. The sub-images of three datasets with different densities are shown in **Figure 4**.

### Model improvement

In the image, the pigment glands occupy a small area, so the object detection method is more challenging in locating the bounding box than the large and mesoscale objects. In the prediction process, if the prediction bounding box is shifted by one pixel, the impact on the small object will be much higher than that on the large and mesoscale object, so it is difficult to achieve high detection accuracy. The method of image segmentation is to classify each pixel in the image at pixel level. So, we used a semantic segmentation model based on deep learning to classify the leave images into pigment glands and non-pigment glands at pixel level. Compared with the manual object detection method, the label map generated by semantic segmentation was simpler, and thus easier to stitch the sub-images together, and the context information was enhanced better.

U-Net was originally used in medical image segmentation. Because the network structure fused the deep features and shallow features with skip connections, U-Net is more effective in dealing with the complex segmentation. U-Net is a process of encoding and decoding, mainly composed of contracting path, expansive path and prediction network (Ronneberger et al., 2015). The contracting path is composed of five effective feature layers, and each of them performs two 3×3 convolution operations and one 2×2 maximum pooling operation for feature extraction. The structure of extraction is to reduce the feature images. After a series of convolution and pooling operations, the size of the feature image was halved and the number of channels increased. The expansive path is symmetric with that of the contracting path, and each effective feature layer was subjected to two 3×3 convolutions and one 2×2 deconvolution operation, that is to say, the five effective feature layers obtained from the feature extraction network were for up-sampling to magnify the reduced image. Finally, the corresponding effective feature layers in the contracting path and the expansive path were spliced and fused through skip connections. The fused feature layer combines the complex information extracted from the deep network and some simple information from the shallow network, such as edge feature, so that the network can handle more complex segmentation tasks. Then the final feature layer fused during feature extraction and enhanced feature extraction was predicted, and the input images were classified on the pixel level. During prediction, the number of channels was converted once, resulting in the number of output channels to be the number of categories classified.

**Figure 5:**
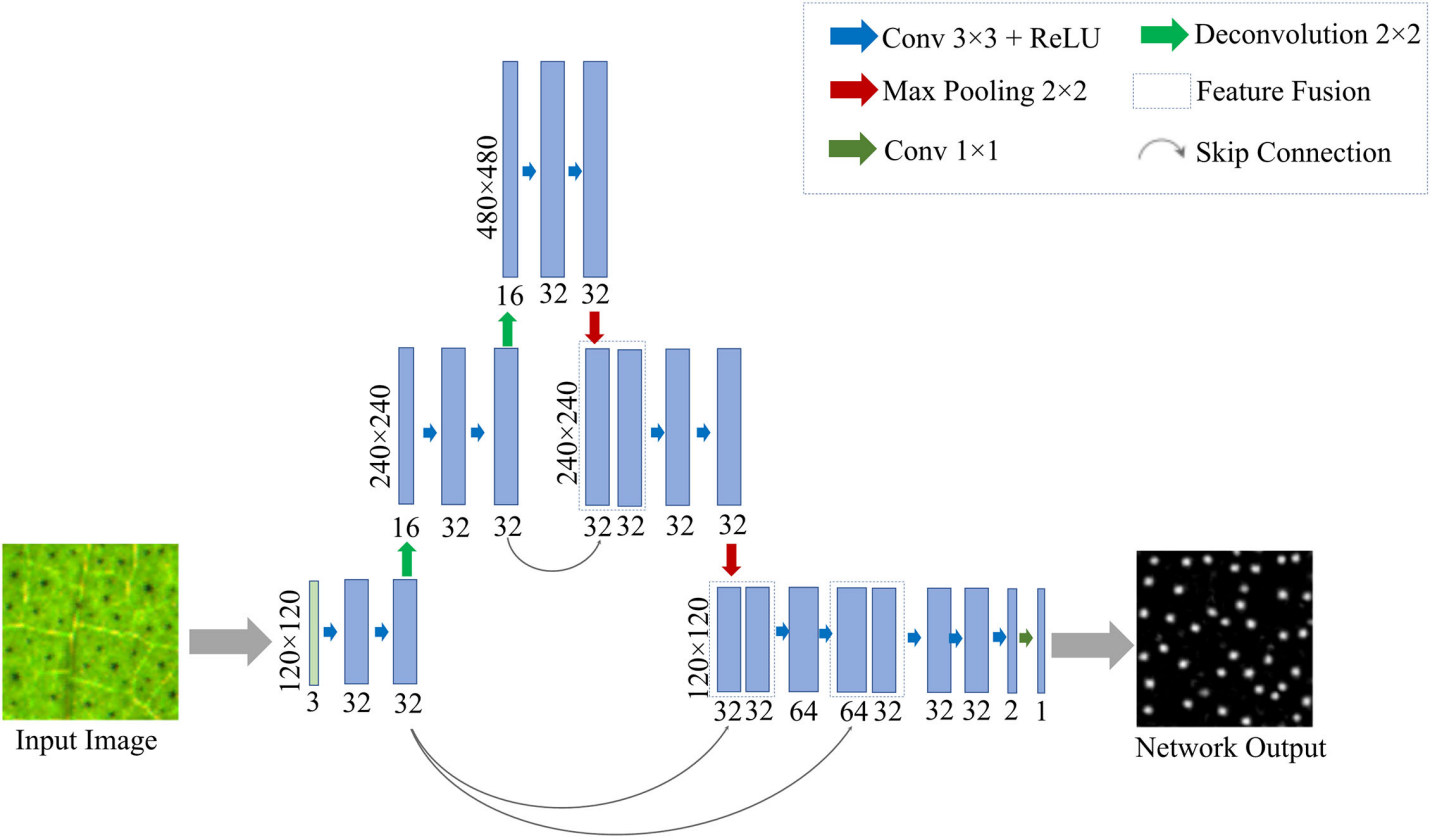
Ipp Net model. The structure of up-sampling and down-sampling is adopted, and the up-sampling and down-sampling are performed twice respectively to obtain image features.

In this experiment, the cotton pigment glands were the only a few pixels in the leaf image. The cotton leaf image will be downscaled by half every time while U-Net model was down-sampled, making feature extraction more difficult, and the feature loss more serious during the training of the model and thus impair the successful realization of cotton pigment glands segmentation. We inverted the classic U-Net structure with a conical structure of interpolation followed by pooling instead of the original “U” structure of down-sampling followed by up-sampling, and the improved network model was named interpolation-pooling net (abbreviated as Ipp Net). This network structure can effectively suppress the loss of pigment gland features caused by the pooling operations. The improved network model has three chief components, up-sampling, down-sampling and network prediction. The Ipp Net model structure is shown in **Figure 5**.

Up-sampling. The sub-images of cotton leaves were entered into the model as input. After two 3×3 convolution layers and batch standardization, the nonlinear ability was strengthened through the ReLU activation function. Then the nearest-neighbor interpolation method was used to amplify the image features. The nearest pixel among the 4 pixels around the pixel to be interpolated was selected as the target pixel and inserted into the amplified image, and the final image was amplified from the original 120×120 to 240×240, and the number of channels increased to 16. After convolutions and interpolation operation, the feature image size was doubled again to 480×480, and the number of channels remained unchanged. For each convolution and interpolation operation, the image size doubled once. The un-sampling process of feature extraction was to enlarge the feature map. Unlike the traditional U-Net model, the improved Ipp Net adopts the Same convolution. The size of the convolution kernel won’t affect the size of the feature map after convolution, but not the step size used for convolution, which was set as 1. The result shows that the size of the output feature map after convolution was the same as the input image. In this mode, the size of the feature image is unchanged during forward propagation, and the convolution operation does not need to accurately calculate the size of the image.

Down-sampling. The feature extraction after the feature images were amplified was performed using convolution and max pooling operations. The pooling method used max pooling to retain the salient features of the image and reduce the feature dimension, so that the model can learn the edge and texture structure of pigment glands, and stably segment the phenotypic traits of pigment glands. After two convolutions and one pooling, the salient features of the image were retained, and the image size was reduced from 480×480 to 240×240. Then, two more convolutions and one pooling operation are performed, and finally reduced to the size of the input image. After each set of convolution and pooling operations, the size of the feature image was reduced to half of the original size. The improved network is also a cross-layer connection, the Concat dimension splicing and fusion method are adopted to fuse the primary features corresponding to the up-sampling and the down-sampling, and the channel number was also increased the same as the corresponding feature layer. On the one hand, the fused feature layer recovered the lost image position information during down-sampling, on the other hand, the low-level details from different scale feature maps were combined with high-level semantics, which enriched the feature information.

Network prediction. The last layer of the network is a 1×1 convolution layer. The feature vector can be converted into the number of required classification results in the layer. The results in the output layer of the network are transformed into nonlinear values by ReLU activation function, that is, the values less than 0 become 0, and the values greater than 0 are assigned as 1, which is used for the conversion of discrete probability values to binary. Finally, the output is single-channel.

### Image cropping and stitching

**Figure 6:**
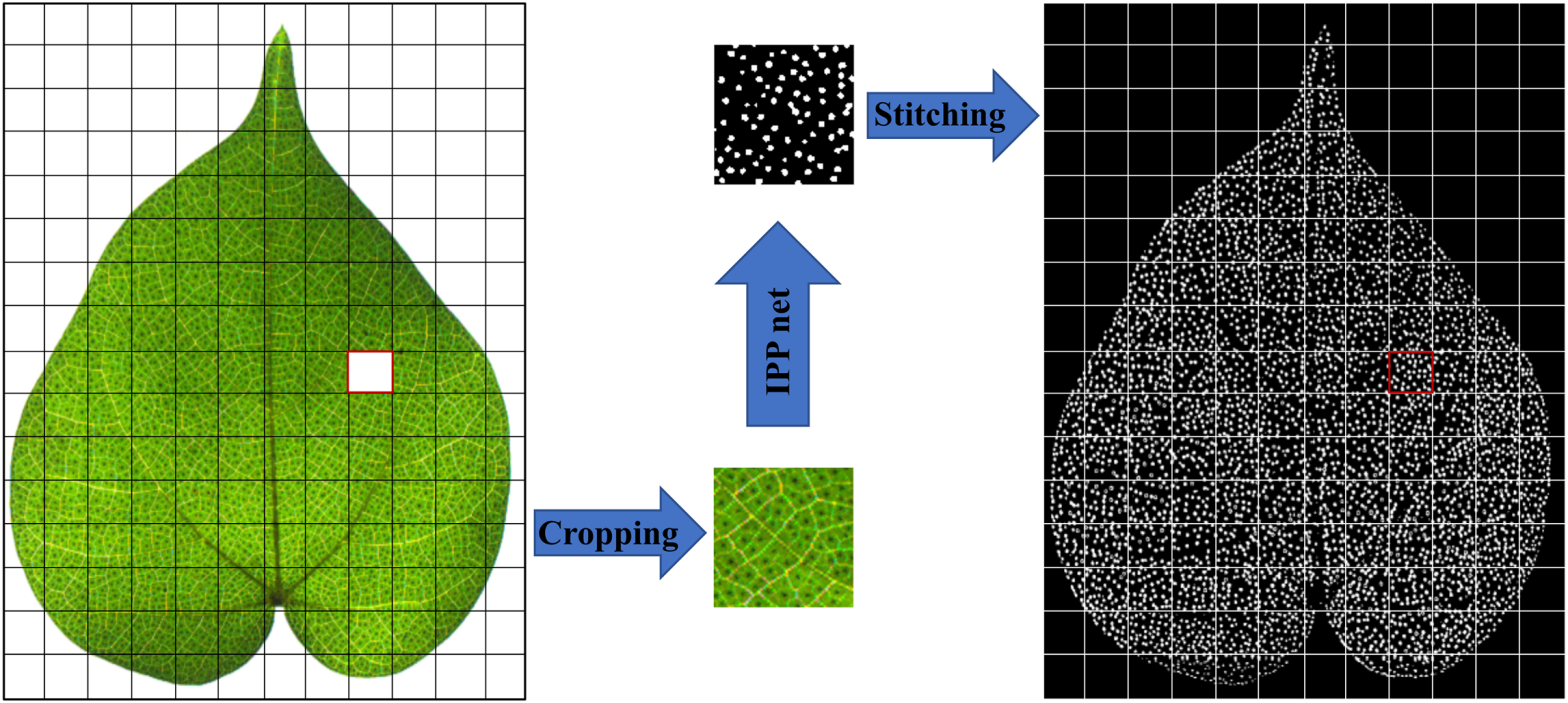
Image cropping and stitching. The red box was one of the sub-images, which has been cropped and segmented and then stitched.

Since the leaf image contains a wide-angle view, which makes GPU memory allocation difficult, and also because that the global semantic information of the pigment gland is not so important in the pigment gland segmentation, even a portion of an intact leaf is taken out, the pigment gland can still be identified. Therefore, we cropped each image into sub-images with the size of 100×100, as shown in **Figure 6**, and a total of 6912 sub-images were cropped. Image cropping reduced the GPU pressure on the one hand and expanded the data set on the other hand. However, in the process of stitching, we found that the undetectable white stripe will appear on the edge. To identify it, we expanded the original sub-image of 100×100 to 120×120 and fed into the network. When the part of 4×5 pixels on the sub-image edge was cropped off, and the remaining images were stitched, which effectively suppressed the impact of edge defects on segmentation, and reduced the counting error caused by incomplete pigment glands at the cropped edges.

## Result

### Model parameter

Python 3.6 was used as the server environment for network training to train and test the model under TensorFlow 1.14.0 and CUDA 10.0. The server was equipped with NVIDIA GeForce GTX 1080Ti graphics processing unit for acceleration and 16GB video memory.

Mean square error (MSE) is used as the loss function of the model to evaluate the difference between the predicted value and the real value of the model, so as to provide the model with an object that can be optimized and make the optimizer move in the right direction.


(1) $$\text{MSELoss}=\frac{1}{n}\sum_{k}{\left(y_{k}-t_{k}\right)}^{2}$$

where *y*_*k*_ s the output of the network, *t*_*k*_  the label value of training data, and *k* represents the size of the training set, *n* is the number of samples.

To make each parameter of the loss function reach the most appropriate value and make the loss function as smooth as possible, we used the adaptive motion estimation (Adam) optimizer to dynamically adjust the learning rate of each parameter using the first-order moment estimation and second-order moment estimation of the gradient. Under Adam, the learning rate bias was corrected, and the parameters were more stable and better adapted to the problem of gradient sparsity. The parameter settings of training model are shown in **Table 1**.

**Table 1:** The parameter settings of training model.

Parameter	Value
**optimizer**	adam
**Epochs**	60
**Steps per epoch**	250
**Batch size**	2

### Evaluation indicators

**Figure 7:**
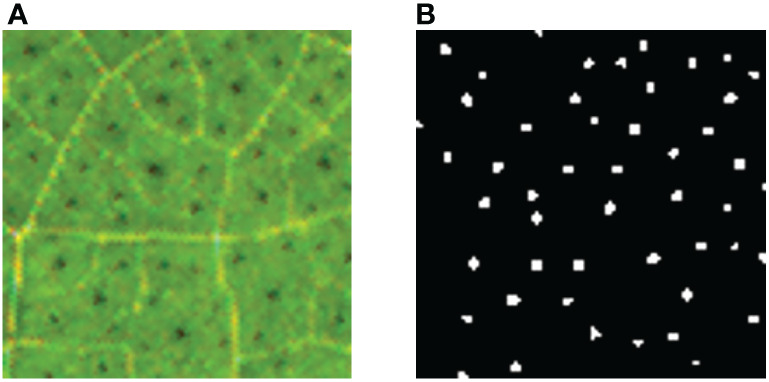
Segmentation results. **(A)** Original image; **(B)** Segmented result.

The segmentation results of the model are shown in **Figure 7**. It is known from **Figure 7** that the model has a good effect on the identification of pigment glands in cotton leaves. In order to objectively and reasonably evaluate the effect of the network model in the pigment gland segmentation of cotton leaves, four model evaluation indicators were introduced to evaluate the segmentation effect of the model and they were mIoU (Mean Intersection over Union), Precision, Recall and F1-score respectively. Four evaluation indicators defined by the equations from (2) to (5) were calculated by the confusion matrix, as shown in **Table 2**, the columns in the table represent the predicted values of the model, and the rows represent the label values of the model.

**Table 2:** Confusion matrix.

P\L	Positive	Negative
Positive	42681	10644
Negative	10645	666030


(2) $$\text{mIoU}=\frac{1}{k+1}\sum_{i=0}^{k}\frac{\text{TP}}{\text{TP}+\text{FP}+\text{FN}}$$

(3) $$\text{Precision}=\frac{\text{TP}}{\text{TP}+\text{FP}}$$

(4) $$\text{Recall}=\frac{\text{TP}}{\text{TP}+\text{FN}}$$

(5) $$\text{F}1-score=\frac{2\times \text{Precision}\times \text{Recall}}{\text{Precision}+\text{Recall}}=\frac{2\times \text{TP}}{2\times \text{TP}+\text{FP}+\text{FN}}$$

Where, True Positive (TP) is the number of the correctly identified pigment gland pixels defined by the model; False Negative (FN) refers to the number of pixels wrongly identified by the model as the background; False Positive (FP) indicates the number of pixels incorrectly identified as the pigment gland, True Negative (TN) is the number of pixels of the background correctly identified as the background.

Mean Intersection over Union (mIoU) is a common evaluation metric for semantic image segmentation, which first computes the IoU for each semantic class and then computes the average over classes. It is a commonly used metric for the measurement of the image segmentation performance of the algorithm at pixel level. It is also used to compare the similarities and differences between the segmentation results and the label set. The Precision indicates that the proportion of pigment glands predicted by the model is close to the actual result. Recall indicates how many positive examples in the sample are predicted correctly. F1-score, also called balanced F score, is defined as the harmonic mean of Precision and Recall. During the model training, mIoU, Recall, Precision and F1-score of each epoch output is calculated in detail using the validation set, followed by the model performance evaluation using the test set not involved in the training.

After each epoch, the accuracy and loss values are calculated in the training set and validation set to monitor the fitting degree of the model. The total model training last about 9.5 h. The loss value of the model appeared to be flat at the 40th epoch and the accuracy of the model stabilized at 0.967; the loss value was finally stabilized at 0.0238, mIoU, Recall, Precision and F1-score was 0.8181, 0.8004, 0.8004 and 0.8004 respectively.

**Figure 8:**
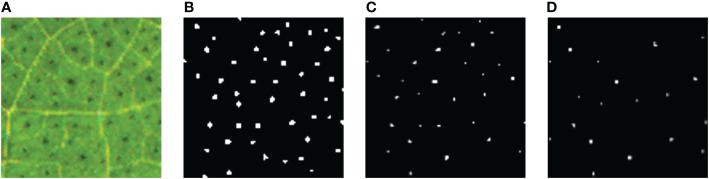
Comparison of segmentation results of the three models. **(A)** Original image; **(B)** The segmentation result of Ipp Net; **(C)** The segmentation result of U-Net; **(D)** The segmentation result of DeepLabv3+.

**Figure 9:**
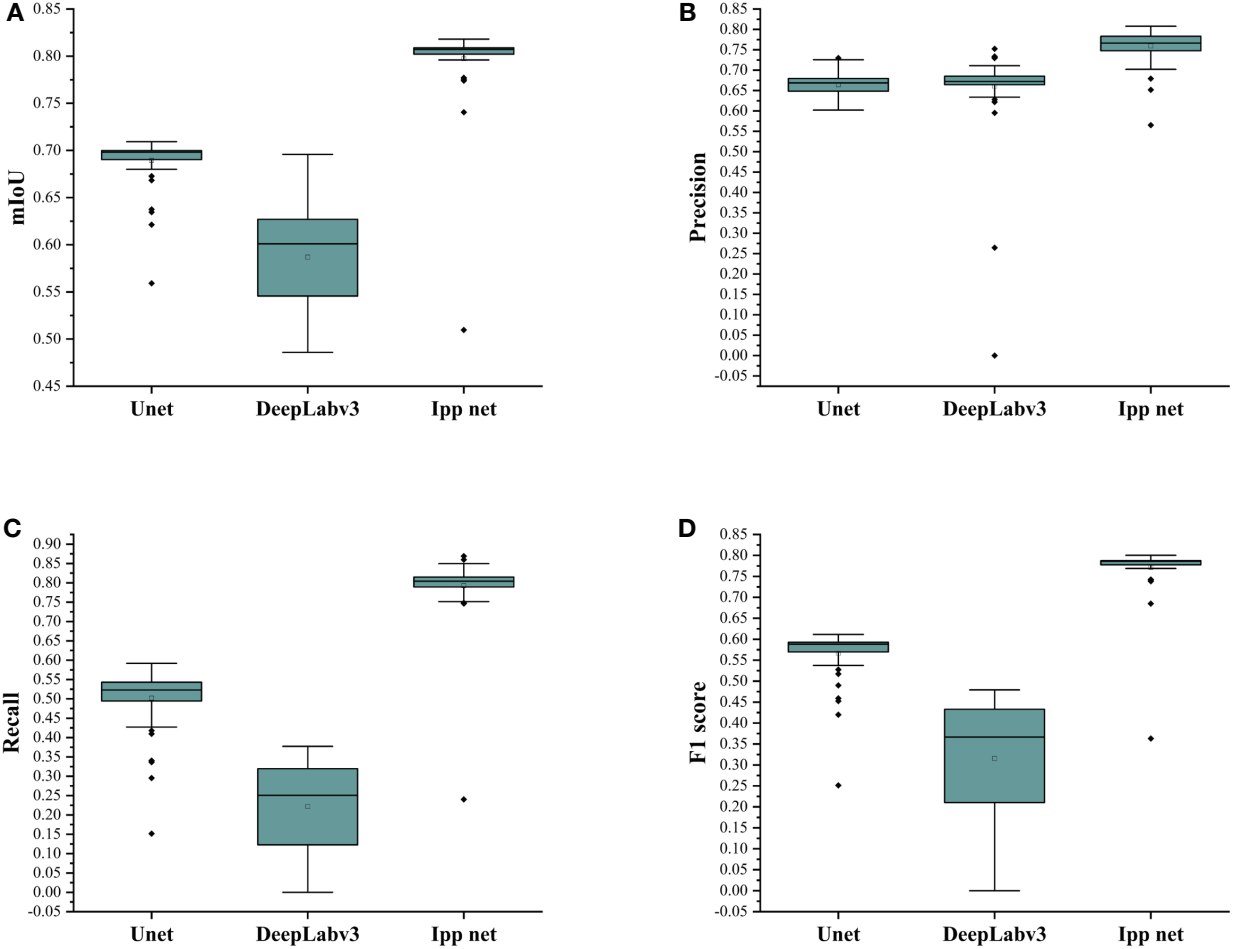
Evaluation indicators of U-Net, DeepLabv3+ and Ipp Net models of different epochs. **(A)** mIoU; **(B)** Precision; **(C)** Recall; **(D)** F1-score.

In addition, two other classical models, U-Net and DeepLabv3+, were trained and compared with the Ipp Net in this paper. The segmentation results are shown in **Figure 8**. The experiment results based on the performance measurement evaluation are shown in **Table 3**. **Figure 9** is the scores of box plot of the three models in the different epochs of the test set. It mainly contains six data nodes, which respectively calculate the upper edge upper quartile Q3, median, lower quartile Q1, lower edge and outliers of test sets in different epochs of mIoU, Recall, Precision and F1-score. Compared with the original U-Net, mIoU, Recall, Precision and F1-score increased by 9.51%, 9.51%, 21.78% and 16.24% respectively, and which was 14.78%, 7.98%, 37.43% and 26.49% better than DeepLabv3+. The segmentation results show that U-Net and DeepLabv3+ are not effective in the segmentation of cotton pigment glands with small objects and dense distribution. In contrast of the model used in this paper, U-Net and DeepLabv3+ will cause the feature loss of pigment glands during feature extraction, so the lost feature information will be classified as background during pixel classification. The model in this paper adopts the structure of interpolation and pooling, which is to amplify the features of the image first and then extract the image features. Therefore, the Ipp Net model has a better segmentation effect in dealing with small objects.

**Table 3:** Evaluation indicators.

Model	Acc	mIoU	Precision	Recall	F1-score
U-Net	0.995	0.723	0.7053	0.5826	0.638
DeepLabv3+	0.994	0.6703	0.7206	0.4261	0.5355
**Ipp Net**	**0.967**	**0.8181**	**0.8004**	**0.8004**	**0.8004**

The bold values are the improved algorithm and corresponding evaluation indicators.

### Results optimization

**Figure 10:**
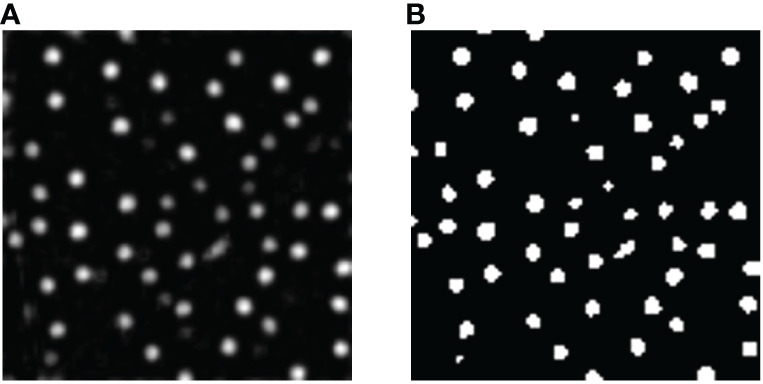
Segmentation results optimization. **(A)** Segmentation results of Ipp Net; **(B)** Optimized images of the segmentation results.

Due to the vein shadows in cotton leaves which are similar to pigment glands, they will be classified as foregrounds, thereby reducing the accuracy of the model. At this time, if the pigment glands are directly counted from the segmentation results, some leaf vein shadows will also be inclusive and counted, and the counting result will be much higher than the actual number of pigment glands. By optimizing the segmentation results, the mistakenly inclusive vein shadows can be filtered, thus weaken the influence of vein shadows on the final counting results. The segmented leaf images were optimized using the machine vision software HALCON 17.12.0.0, and the optimization results are shown in **Figure 10**. Select_shape is an operator that can select the regions according to the shape features such as the area and roundness of the input connected domain. Its usage is simple and has good effect on image filtering and optimization. The diameter of the pigment glands is generally 100~400μm, approximately a few pixels as an image. 3 pixels was the minimum pixel value of the pigment glands in the sample image and we optimized the image with shape feature selection and morphological filtering. The connected domains with area parameters between 2-938 744 pixels were selected and counted, while the connected domains with area parameters less than 2 pixels were filtered out. Then, the count_obj operator was used to count the connected domains selected by the select_shape operator, and the optimized image was saved to count the pigment glands.

The image was optimized by the combination of morphological filtering and shape feature screening, which eliminated the shadow impurities of leaf veins, and at the same time preserved the characteristics of the pigment glands in the image. Compared with other filtering methods, such as median filtering, shape feature screening, this method can well preserve the features of very small pigment glands.

### Counting results

**Figure 11:**
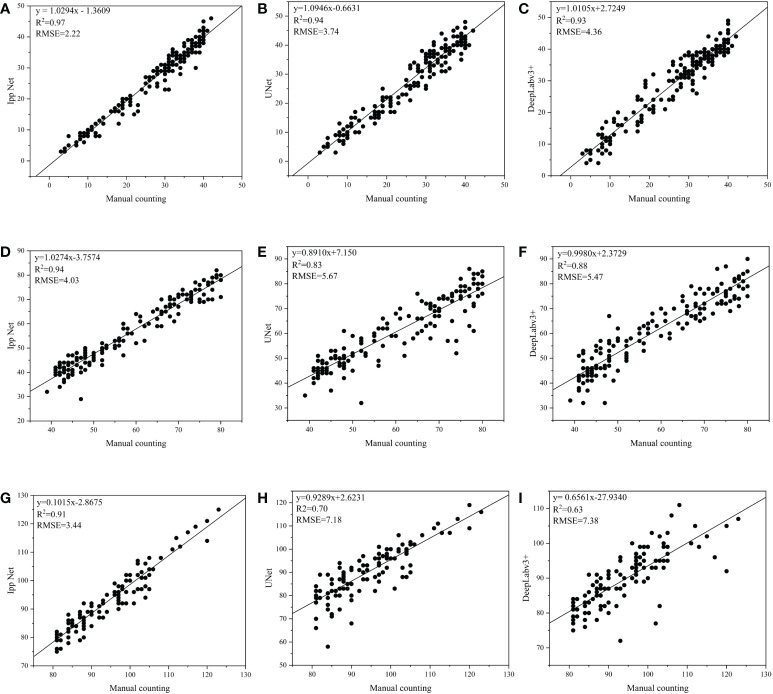
Comparison results of Ipp Net, U-Net and DeepLabv3+ with manual counting at three different densities. **(A–C)**: DS1, low-density; **(D–F)**: DS2, medium-density; **(G–I)**: DS3, high-density.

In order to better evaluate the Ipp Net model, the pigment glands of 453 sub-images in three datasets DS1, DS2 and DS3 used for model evaluation were segmented and counted, **Figure 11** shows the comparison results of Ipp Net, U-Net and DeepLabv3+ with manual counting at three densities. The abscissa in **Figure 11** is the result of manual counting, and the ordinate is the counting result of Ipp Net, U-Net and DeepLabv3+. As shown in **Figure 11**, the square of the correlation coefficient (*R* ^2^) of Ipp Net reached 0.97, 0.94 and 0.91 respectively in the three datasets, DS1, DS2 and DS3, which were 0.03, 0.11, 0.21 and 0.04, 0.06, 0.28 higher than those of U-Net and DeepLabv3+, respectively. With the increase of pigment gland density, the detection and counting ability of the three models decreased, but Ipp Net still performed well in the detection and counting high-density pigment glands, and *R* ^2^ still reached 0.91. The results show that Ipp Net has the best fitting degree and is more accurate than U-Net and DeepLabv3+ at different densities. The proposed method can realize high-throughput detection and counting of cotton pigment glands.

## Discussion

Gossypol is a peculiar substance of Gossypium plants. It is toxic and has extremely high research value in agriculture, pharmaceutics and other fields. At present, the detection of gossypol mainly leverages High Performance Liquid Chromatography (HPLC), spectrophotometry, Capillary Electrophoresis and other chemical reagent methods. These methods require to break up the cotton organ samples for detection. Although the result can achieve high accuracy, they are not economical and non-destructive. Plant phenotypic analysis has made important progress in crop identification and detection. Pigment gland is the main carrier of gossypol. There is a significant correlation between the phenotypic traits of pigment gland and gossypol content, which can be used as a basis for measuring the amount of gossypol in phenolic gossypol. Pigment glands are small and densely distributed. At present, people mostly analyze the distribution of pigment glands by hand, and the workload is huge. To reduce work, we proposed a machine vision method to detect the number of pigment glands in cotton leaves. Compared with the manual method, it is more convenient and detect faster, and the distribution of pigment glands in cotton leaves can be analyzed more accurately.

The densely distributed cotton pigment gland is only a few pixels in the image of cotton leaves, and the detection is counted as small object detection. Due to the small coverage area in the image and hardly available features of small objects that are distributed in a dense manner, it is difficult to locate the general large objects. At the same time, the predicted bounding box may also filter out a large number of correct ones due to the non-maximum suppression operation during post-processing, resulting in overlooking detection. Therefore, some commonly used convolutional neural networks cannot achieve good detection results for the detection of dense small objects. The U-Net network performs feature fusion of different dimensions in the channel dimension for the network to segment and detect images of different sizes. In the middle of detection, the object will be aggregated at one point in the feature image after multiple times of down-samplings, which makes the feature extraction more difficult. Therefore, the detection effect on the cotton leaf pigment gland using the U-Net network is not accurate.

In this paper, a semantic segmentation model based on interpolation-pooling network is proposed to materialize automatic segmentation of cotton pigment glands, and count the segmentation results. The improved Ipp Net is in the sequence of interpolation first and then pooling to avoid the target feature information loss caused by down-sampling in the classic U-Net network structure, and improve the detection accuracy of the network for small objects. The experiment results show that the improved Ipp Net has high segmentation accuracy of 0.967 for the cotton leaf image data set. In order to verify the ability of Ipp Net to detect the pigment glands with different densities, three data sets were made according to the density distribution of pigment glands in cotton leaf sub-images to test the counting results of the model. Compared with the manual counting methods, the detection accuracy of Ipp Net is slightly lower than that of manual counting, but the required detection time is shorter and labor is saved. For the trained model, the average detection time of each sub-image is only 202 ms, and it takes about 91.5 s to detect the 453 sub-images, while the manual method takes about 11 hours to complete the statistical task of pigment glands. Therefore, the semantic segmentation model proposed in this paper can replace the manual method for counting the cotton pigment glands.

Different from other small object detection methods, we used the semantic segmentation method to detect the pigment glands, and used the object detection method to detect small objects with very dense distribution. After sampling for several times, the small objects adjacent to the aggregation area would be aggregated together in the deep feature map, resulting in the model being difficult to distinguish. The boundary distance between small objects in the aggregation area is too close, which will lead to the difficulty of bounding box regression and the difficulty of model convergence. It is difficult to achieve the ideal detection effect. The method of semantic segmentation is to classify each pixel in the image, so that the detection of pigment glands is more accurate, and the counting result of pigment glands is closer to the real value.

Before constructing the Ipp Net model, we used the traditional image segmentation methods such as threshold segmentation to detect the pigment glands in cotton leaves. However, due to the small size and dense distribution, the traditional image segmentation method was not ideal for the segmentation of pigment glands. Especially in some images containing veins and leaf edges, the detection results were too different from the real values, and the pigment gland counting task cannot be completed at all. In addition, the pigment glands in the image were basically unrecognized in the intact leaves with a wide field of view.

**Figure 12:**
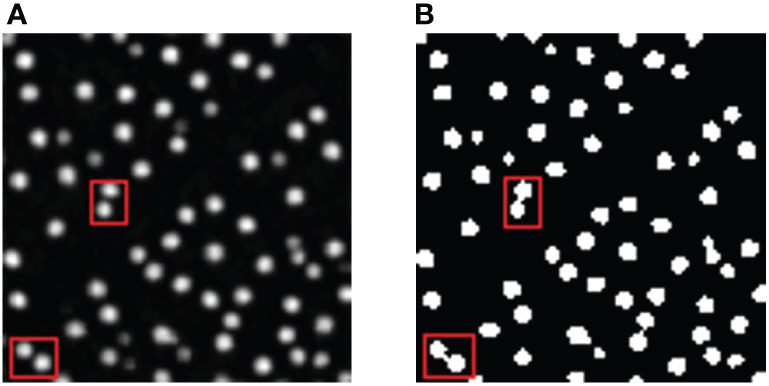
Cases of misjudgment. **(A)** The segmentation result of Ipp Net model; **(B)** The result after filtering and optimization. The red box is the part of the image optimized to cause the adhesion of pigment glands.

However, the model still suffers from false detection of pigment glands. The convolutional neural network model is easily affected by the quality of image annotation. The main reason for that is because of the veins and leaf edges in cotton leaves. When labelling the pigment glands on the leaf veins, some of the veins will also be annotated. In this way, the model will also learn from wrong labelling. Affected by the white light, the edges of the leaf were too bright, making it more difficult to mark manually, so the leaf edge will also cause false detection. In addition, the color of the pigment gland was very close to vein shadow, making it even harder for the computer to distinguish. Consequently, some vein shadows are mistakenly identified as pigment glands in the model, thus reducing the accuracy of the model. When the semantic segmentation of general objects is carried out in neural network, the edges of the object in the image have been through pixel mutation and contain richer feature information than the inside of the object, so it is easier to recognize them. The pigment glands have fuzzy edges which contain very little information. Although the pigment glands in the image are easy to identify, the edge segmentation is usually not ideal. On the other hand, the closing operation of morphological filtering is used for image optimization, which leads to some adjacent pigment glands forming a connected domain of adhesion after closing operation. As shown in **Figure 12**, A is the leaf image segmented by the improved Ipp Net, and B is the optimized image, and the counting result will be smaller than the true value.

Image enhancement of the data set is an important method to improve the accuracy of convolutional neural network. Illumination and noise in the imaging process are important influential factors on the detection accuracy of the model. Although the IPP Net model has a good effect in identifying the pigment glands in cotton leaves, its segmentation effect is easily affected by the veins shadow of cotton leaves and the image noise of leaf edge. The results were optimized by shape feature filtering, but there is still some gap between the automatic counting results and the true value. In the follow-up research, on the one hand, the cotton leaf data set was smoothed and filtered to eliminate the influence of veins and their shadows on the model accuracy as much as possible while retaining the characteristics of pigment glands; on the other hand, we will proceed to further improve the model and try to integrate the global context information into the detection model, so as to improve the detection accuracy of the model in the veins to minimize the influence of vein shadow. In addition, the function to calculate the area of pigment glands and to estimate the content of Gossypol in cotton leaves by combining the area and quantity will be added to the model. A non-destructive and accurate estimation model of Gossypol content will be constructed, and then the model of cotton disease resistance, insect resistance, gland phenotypic traits and physical and chemical parameters of Gossypol content was established to realize the auxiliary decision-making technology of disease resistance and insect resistance quality identification.

## Conclusions

Cotton pigment gland is the main carrier of gossypol, an important structure for studying gossypol and its derivatives. Its density and size reflect the amount of gossypol in glanded cotton. Cotton pigment glands are extremely small and densely distributed. At present, researchers mostly use microscope observation to estimate the density of pigment glands based on artificial experience. The manual counting method is easily influenced by the experience of researchers and is time-consuming and laborious, which brings great inconvenience to the study of cotton pigment glands and gossypol.

In this paper, a neural network model for automatic detection and counting of pigment glands in cotton leaves is proposed, aiming to detect small and densely distributed pigment glands in cotton leaves by computing of the semantic segmentation model. The interpolation-pooling network is proposed, and the practice of interpolation first and then pooling can effectively avoid the loss of target feature information caused by convolution, which is more conducive to the extraction of small objects information with small individual or small proportion in the image. The model was validated using the images of glanded cotton true leaves, and mIoU, Recall, Precision and F1-score were used to evaluate the network performance. The final validation set scores were 0.8181, 0.8004, 0.8004 and 0.8004 respectively. Compared with U-Net and DeepLabv3+, the mIoU, Recall, Precision and F1-score of Ipp Net are higher by 9.51%, 9.51%, 21.78%, 16.24% and 14.78%, 7.98%, 37.43% and 26.49% respectively. The segmentation has better outcome. In addition, the detection ability of the Ipp Net model in cotton pigment glands of different densities was analyzed, and found that it has good performance in different densities. The results show that the Ipp Net model has better segmentation effect and good robustness, and can replace manual counting for cotton pigment gland counting in some researches, which provides an important practical reference for the study of cotton pigment gland.

## Data availability statement

The original contributions presented in the study are included in the article/**Supplementary Material**. Further inquiries can be directed to the corresponding author.

## Author contributions

LShe, NW and LShao conceived the idea and proposed the method. LShe, YX, and GW contributed to the preparation of equipment and acquisition of data. LShe, YX, and NW wrote the code and tested the method. LShe and YX validated the results. LShe wrote the manuscript. LShe, NW and LShao revised the manuscript. All authors contributed to the article and approved the submitted version.
