# Supplementary figures and images for: Detection and counting of pigment glands in cotton leaves using improved U-Net (part 2 of 2)
Source: Front Plant Sci. 2023 Jan 9;13:1075051. doi: 10.3389/fpls.2022.1075051 (PMC9869271; doi:10.3389/fpls.2022.1075051)

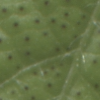

Supplement: Supplementary file 1 [file DataSheet_1.zip › net_for_leaf/data/mydata11/train/image1/1/1066.png]

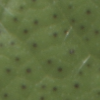

Supplement: Supplementary file 1 [file DataSheet_1.zip › net_for_leaf/data/mydata11/train/image1/1/1067.png]

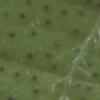

Supplement: Supplementary file 1 [file DataSheet_1.zip › net_for_leaf/data/mydata11/train/image1/1/1068.png]

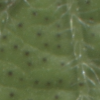

Supplement: Supplementary file 1 [file DataSheet_1.zip › net_for_leaf/data/mydata11/train/image1/1/1069.png]

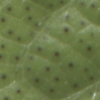

Supplement: Supplementary file 1 [file DataSheet_1.zip › net_for_leaf/data/mydata11/train/image1/1/107.png]

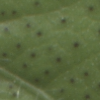

Supplement: Supplementary file 1 [file DataSheet_1.zip › net_for_leaf/data/mydata11/train/image1/1/1070.png]

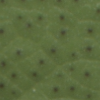

Supplement: Supplementary file 1 [file DataSheet_1.zip › net_for_leaf/data/mydata11/train/image1/1/1071.png]

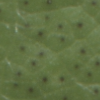

Supplement: Supplementary file 1 [file DataSheet_1.zip › net_for_leaf/data/mydata11/train/image1/1/1072.png]

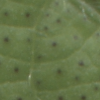

Supplement: Supplementary file 1 [file DataSheet_1.zip › net_for_leaf/data/mydata11/train/image1/1/1073.png]

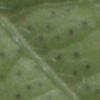

Supplement: Supplementary file 1 [file DataSheet_1.zip › net_for_leaf/data/mydata11/train/image1/1/1074.png]

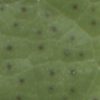

Supplement: Supplementary file 1 [file DataSheet_1.zip › net_for_leaf/data/mydata11/train/image1/1/1075.png]

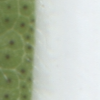

Supplement: Supplementary file 1 [file DataSheet_1.zip › net_for_leaf/data/mydata11/train/image1/1/1076.png]

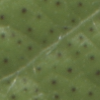

Supplement: Supplementary file 1 [file DataSheet_1.zip › net_for_leaf/data/mydata11/train/image1/1/108.png]

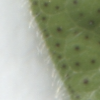

Supplement: Supplementary file 1 [file DataSheet_1.zip › net_for_leaf/data/mydata11/train/image1/1/1080.png]

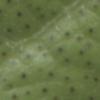

Supplement: Supplementary file 1 [file DataSheet_1.zip › net_for_leaf/data/mydata11/train/image1/1/1081.png]

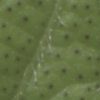

Supplement: Supplementary file 1 [file DataSheet_1.zip › net_for_leaf/data/mydata11/train/image1/1/1082.png]

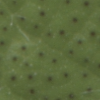

Supplement: Supplementary file 1 [file DataSheet_1.zip › net_for_leaf/data/mydata11/train/image1/1/1083.png]

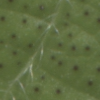

Supplement: Supplementary file 1 [file DataSheet_1.zip › net_for_leaf/data/mydata11/train/image1/1/1084.png]

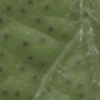

Supplement: Supplementary file 1 [file DataSheet_1.zip › net_for_leaf/data/mydata11/train/image1/1/1085.png]

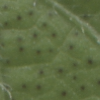

Supplement: Supplementary file 1 [file DataSheet_1.zip › net_for_leaf/data/mydata11/train/image1/1/1086.png]

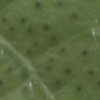

Supplement: Supplementary file 1 [file DataSheet_1.zip › net_for_leaf/data/mydata11/train/image1/1/1087.png]

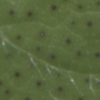

Supplement: Supplementary file 1 [file DataSheet_1.zip › net_for_leaf/data/mydata11/train/image1/1/1088.png]

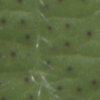

Supplement: Supplementary file 1 [file DataSheet_1.zip › net_for_leaf/data/mydata11/train/image1/1/1089.png]

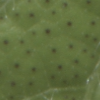

Supplement: Supplementary file 1 [file DataSheet_1.zip › net_for_leaf/data/mydata11/train/image1/1/109.png]

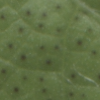

Supplement: Supplementary file 1 [file DataSheet_1.zip › net_for_leaf/data/mydata11/train/image1/1/1090.png]

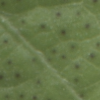

Supplement: Supplementary file 1 [file DataSheet_1.zip › net_for_leaf/data/mydata11/train/image1/1/1091.png]

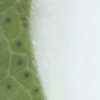

Supplement: Supplementary file 1 [file DataSheet_1.zip › net_for_leaf/data/mydata11/train/image1/1/1092.png]

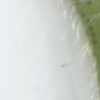

Supplement: Supplementary file 1 [file DataSheet_1.zip › net_for_leaf/data/mydata11/train/image1/1/1096.png]

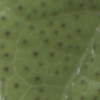

Supplement: Supplementary file 1 [file DataSheet_1.zip › net_for_leaf/data/mydata11/train/image1/1/1097.png]

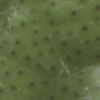

Supplement: Supplementary file 1 [file DataSheet_1.zip › net_for_leaf/data/mydata11/train/image1/1/1098.png]

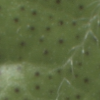

Supplement: Supplementary file 1 [file DataSheet_1.zip › net_for_leaf/data/mydata11/train/image1/1/1099.png]

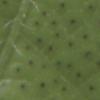

Supplement: Supplementary file 1 [file DataSheet_1.zip › net_for_leaf/data/mydata11/train/image1/1/110.png]

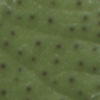

Supplement: Supplementary file 1 [file DataSheet_1.zip › net_for_leaf/data/mydata11/train/image1/1/1100.png]

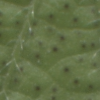

Supplement: Supplementary file 1 [file DataSheet_1.zip › net_for_leaf/data/mydata11/train/image1/1/1101.png]

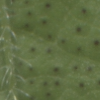

Supplement: Supplementary file 1 [file DataSheet_1.zip › net_for_leaf/data/mydata11/train/image1/1/1102.png]

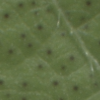

Supplement: Supplementary file 1 [file DataSheet_1.zip › net_for_leaf/data/mydata11/train/image1/1/1103.png]

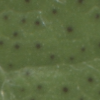

Supplement: Supplementary file 1 [file DataSheet_1.zip › net_for_leaf/data/mydata11/train/image1/1/1104.png]

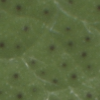

Supplement: Supplementary file 1 [file DataSheet_1.zip › net_for_leaf/data/mydata11/train/image1/1/1105.png]

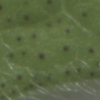

Supplement: Supplementary file 1 [file DataSheet_1.zip › net_for_leaf/data/mydata11/train/image1/1/1106.png]
