# Supplementary figures and images for: Detection and counting of pigment glands in cotton leaves using improved U-Net (part 1 of 2)
Source: Front Plant Sci. 2023 Jan 9;13:1075051. doi: 10.3389/fpls.2022.1075051 (PMC9869271; doi:10.3389/fpls.2022.1075051)

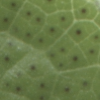

Supplement: Supplementary file 1 [file DataSheet_1.zip › net_for_leaf/data/mydata11/test/0.png]

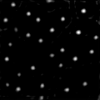

Supplement: Supplementary file 1 [file DataSheet_1.zip › net_for_leaf/data/mydata11/test/0_predict.png]

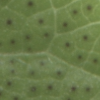

Supplement: Supplementary file 1 [file DataSheet_1.zip › net_for_leaf/data/mydata11/test/1.png]

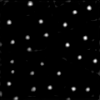

Supplement: Supplementary file 1 [file DataSheet_1.zip › net_for_leaf/data/mydata11/test/1_predict.png]

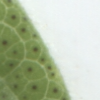

Supplement: Supplementary file 1 [file DataSheet_1.zip › net_for_leaf/data/mydata11/test/2.png]

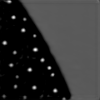

Supplement: Supplementary file 1 [file DataSheet_1.zip › net_for_leaf/data/mydata11/test/2_predict.png]

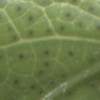

Supplement: Supplementary file 1 [file DataSheet_1.zip › net_for_leaf/data/mydata11/test/65.png]

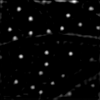

Supplement: Supplementary file 1 [file DataSheet_1.zip › net_for_leaf/data/mydata11/test/65_predict.png]

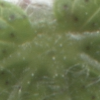

Supplement: Supplementary file 1 [file DataSheet_1.zip › net_for_leaf/data/mydata11/test/66.png]

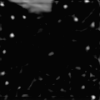

Supplement: Supplementary file 1 [file DataSheet_1.zip › net_for_leaf/data/mydata11/test/66_predict.png]

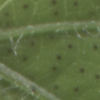

Supplement: Supplementary file 1 [file DataSheet_1.zip › net_for_leaf/data/mydata11/test/67.png]

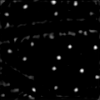

Supplement: Supplementary file 1 [file DataSheet_1.zip › net_for_leaf/data/mydata11/test/67_predict.png]

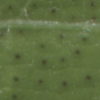

Supplement: Supplementary file 1 [file DataSheet_1.zip › net_for_leaf/data/mydata11/test/68.png]

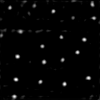

Supplement: Supplementary file 1 [file DataSheet_1.zip › net_for_leaf/data/mydata11/test/68_predict.png]

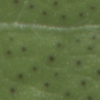

Supplement: Supplementary file 1 [file DataSheet_1.zip › net_for_leaf/data/mydata11/test/69.png]

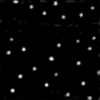

Supplement: Supplementary file 1 [file DataSheet_1.zip › net_for_leaf/data/mydata11/test/69_predict.png]

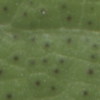

Supplement: Supplementary file 1 [file DataSheet_1.zip › net_for_leaf/data/mydata11/test/70.png]

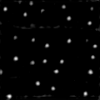

Supplement: Supplementary file 1 [file DataSheet_1.zip › net_for_leaf/data/mydata11/test/70_predict.png]

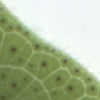

Supplement: Supplementary file 1 [file DataSheet_1.zip › net_for_leaf/data/mydata11/test/72.png]

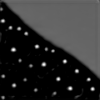

Supplement: Supplementary file 1 [file DataSheet_1.zip › net_for_leaf/data/mydata11/test/72_predict.png]

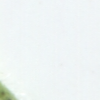

Supplement: Supplementary file 1 [file DataSheet_1.zip › net_for_leaf/data/mydata11/test/73.png]

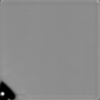

Supplement: Supplementary file 1 [file DataSheet_1.zip › net_for_leaf/data/mydata11/test/73_predict.png]

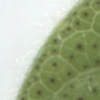

Supplement: Supplementary file 1 [file DataSheet_1.zip › net_for_leaf/data/mydata11/test/77.png]

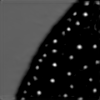

Supplement: Supplementary file 1 [file DataSheet_1.zip › net_for_leaf/data/mydata11/test/77_predict.png]

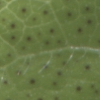

Supplement: Supplementary file 1 [file DataSheet_1.zip › net_for_leaf/data/mydata11/test/78.png]

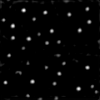

Supplement: Supplementary file 1 [file DataSheet_1.zip › net_for_leaf/data/mydata11/test/78_predict.png]

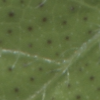

Supplement: Supplementary file 1 [file DataSheet_1.zip › net_for_leaf/data/mydata11/test/79.png]

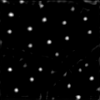

Supplement: Supplementary file 1 [file DataSheet_1.zip › net_for_leaf/data/mydata11/test/79_predict.png]

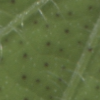

Supplement: Supplementary file 1 [file DataSheet_1.zip › net_for_leaf/data/mydata11/test/80.png]

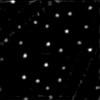

Supplement: Supplementary file 1 [file DataSheet_1.zip › net_for_leaf/data/mydata11/test/80_predict.png]

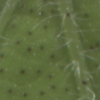

Supplement: Supplementary file 1 [file DataSheet_1.zip › net_for_leaf/data/mydata11/test/81.png]

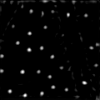

Supplement: Supplementary file 1 [file DataSheet_1.zip › net_for_leaf/data/mydata11/test/81_predict.png]

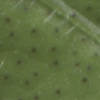

Supplement: Supplementary file 1 [file DataSheet_1.zip › net_for_leaf/data/mydata11/test/82.png]

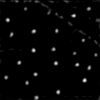

Supplement: Supplementary file 1 [file DataSheet_1.zip › net_for_leaf/data/mydata11/test/82_predict.png]

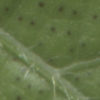

Supplement: Supplementary file 1 [file DataSheet_1.zip › net_for_leaf/data/mydata11/test/83.png]

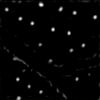

Supplement: Supplementary file 1 [file DataSheet_1.zip › net_for_leaf/data/mydata11/test/83_predict.png]

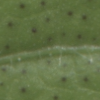

Supplement: Supplementary file 1 [file DataSheet_1.zip › net_for_leaf/data/mydata11/test/84.png]

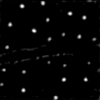

Supplement: Supplementary file 1 [file DataSheet_1.zip › net_for_leaf/data/mydata11/test/84_predict.png]

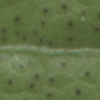

Supplement: Supplementary file 1 [file DataSheet_1.zip › net_for_leaf/data/mydata11/test/85.png]

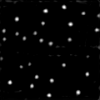

Supplement: Supplementary file 1 [file DataSheet_1.zip › net_for_leaf/data/mydata11/test/85_predict.png]

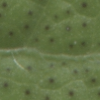

Supplement: Supplementary file 1 [file DataSheet_1.zip › net_for_leaf/data/mydata11/test/86.png]

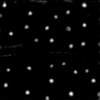

Supplement: Supplementary file 1 [file DataSheet_1.zip › net_for_leaf/data/mydata11/test/86_predict.png]

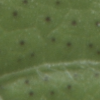

Supplement: Supplementary file 1 [file DataSheet_1.zip › net_for_leaf/data/mydata11/train/image1/1/100.png]

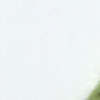

Supplement: Supplementary file 1 [file DataSheet_1.zip › net_for_leaf/data/mydata11/train/image1/1/1000.png]

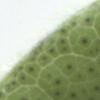

Supplement: Supplementary file 1 [file DataSheet_1.zip › net_for_leaf/data/mydata11/train/image1/1/1001.png]

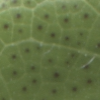

Supplement: Supplementary file 1 [file DataSheet_1.zip › net_for_leaf/data/mydata11/train/image1/1/1002.png]

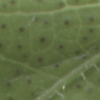

Supplement: Supplementary file 1 [file DataSheet_1.zip › net_for_leaf/data/mydata11/train/image1/1/1003.png]

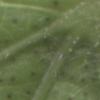

Supplement: Supplementary file 1 [file DataSheet_1.zip › net_for_leaf/data/mydata11/train/image1/1/1004.png]

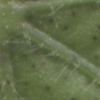

Supplement: Supplementary file 1 [file DataSheet_1.zip › net_for_leaf/data/mydata11/train/image1/1/1005.png]

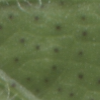

Supplement: Supplementary file 1 [file DataSheet_1.zip › net_for_leaf/data/mydata11/train/image1/1/1006.png]

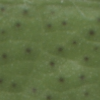

Supplement: Supplementary file 1 [file DataSheet_1.zip › net_for_leaf/data/mydata11/train/image1/1/1007.png]

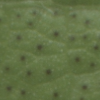

Supplement: Supplementary file 1 [file DataSheet_1.zip › net_for_leaf/data/mydata11/train/image1/1/1008.png]

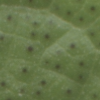

Supplement: Supplementary file 1 [file DataSheet_1.zip › net_for_leaf/data/mydata11/train/image1/1/1009.png]

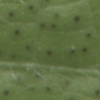

Supplement: Supplementary file 1 [file DataSheet_1.zip › net_for_leaf/data/mydata11/train/image1/1/101.png]

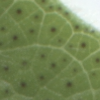

Supplement: Supplementary file 1 [file DataSheet_1.zip › net_for_leaf/data/mydata11/train/image1/1/1010.png]

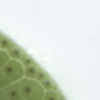

Supplement: Supplementary file 1 [file DataSheet_1.zip › net_for_leaf/data/mydata11/train/image1/1/1011.png]

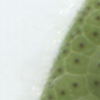

Supplement: Supplementary file 1 [file DataSheet_1.zip › net_for_leaf/data/mydata11/train/image1/1/1016.png]

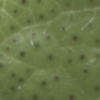

Supplement: Supplementary file 1 [file DataSheet_1.zip › net_for_leaf/data/mydata11/train/image1/1/1017.png]

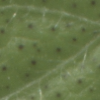

Supplement: Supplementary file 1 [file DataSheet_1.zip › net_for_leaf/data/mydata11/train/image1/1/1018.png]

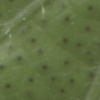

Supplement: Supplementary file 1 [file DataSheet_1.zip › net_for_leaf/data/mydata11/train/image1/1/1019.png]

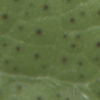

Supplement: Supplementary file 1 [file DataSheet_1.zip › net_for_leaf/data/mydata11/train/image1/1/102.png]

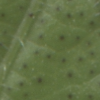

Supplement: Supplementary file 1 [file DataSheet_1.zip › net_for_leaf/data/mydata11/train/image1/1/1020.png]

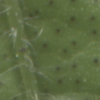

Supplement: Supplementary file 1 [file DataSheet_1.zip › net_for_leaf/data/mydata11/train/image1/1/1021.png]

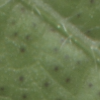

Supplement: Supplementary file 1 [file DataSheet_1.zip › net_for_leaf/data/mydata11/train/image1/1/1022.png]

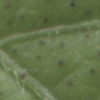

Supplement: Supplementary file 1 [file DataSheet_1.zip › net_for_leaf/data/mydata11/train/image1/1/1023.png]

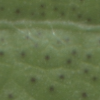

Supplement: Supplementary file 1 [file DataSheet_1.zip › net_for_leaf/data/mydata11/train/image1/1/1024.png]

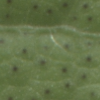

Supplement: Supplementary file 1 [file DataSheet_1.zip › net_for_leaf/data/mydata11/train/image1/1/1025.png]

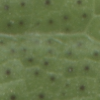

Supplement: Supplementary file 1 [file DataSheet_1.zip › net_for_leaf/data/mydata11/train/image1/1/1026.png]

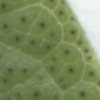

Supplement: Supplementary file 1 [file DataSheet_1.zip › net_for_leaf/data/mydata11/train/image1/1/1027.png]

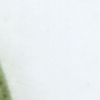

Supplement: Supplementary file 1 [file DataSheet_1.zip › net_for_leaf/data/mydata11/train/image1/1/1028.png]

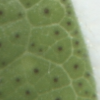

Supplement: Supplementary file 1 [file DataSheet_1.zip › net_for_leaf/data/mydata11/train/image1/1/103.png]

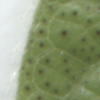

Supplement: Supplementary file 1 [file DataSheet_1.zip › net_for_leaf/data/mydata11/train/image1/1/1032.png]

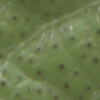

Supplement: Supplementary file 1 [file DataSheet_1.zip › net_for_leaf/data/mydata11/train/image1/1/1033.png]

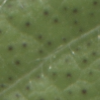

Supplement: Supplementary file 1 [file DataSheet_1.zip › net_for_leaf/data/mydata11/train/image1/1/1034.png]

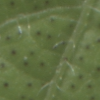

Supplement: Supplementary file 1 [file DataSheet_1.zip › net_for_leaf/data/mydata11/train/image1/1/1035.png]

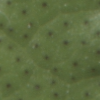

Supplement: Supplementary file 1 [file DataSheet_1.zip › net_for_leaf/data/mydata11/train/image1/1/1036.png]

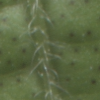

Supplement: Supplementary file 1 [file DataSheet_1.zip › net_for_leaf/data/mydata11/train/image1/1/1037.png]

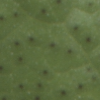

Supplement: Supplementary file 1 [file DataSheet_1.zip › net_for_leaf/data/mydata11/train/image1/1/1038.png]

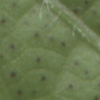

Supplement: Supplementary file 1 [file DataSheet_1.zip › net_for_leaf/data/mydata11/train/image1/1/1039.png]

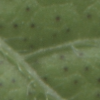

Supplement: Supplementary file 1 [file DataSheet_1.zip › net_for_leaf/data/mydata11/train/image1/1/1040.png]

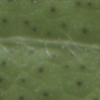

Supplement: Supplementary file 1 [file DataSheet_1.zip › net_for_leaf/data/mydata11/train/image1/1/1041.png]

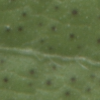

Supplement: Supplementary file 1 [file DataSheet_1.zip › net_for_leaf/data/mydata11/train/image1/1/1042.png]

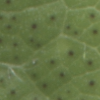

Supplement: Supplementary file 1 [file DataSheet_1.zip › net_for_leaf/data/mydata11/train/image1/1/1043.png]

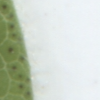

Supplement: Supplementary file 1 [file DataSheet_1.zip › net_for_leaf/data/mydata11/train/image1/1/1044.png]

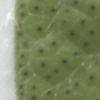

Supplement: Supplementary file 1 [file DataSheet_1.zip › net_for_leaf/data/mydata11/train/image1/1/1048.png]

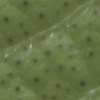

Supplement: Supplementary file 1 [file DataSheet_1.zip › net_for_leaf/data/mydata11/train/image1/1/1049.png]

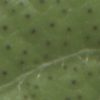

Supplement: Supplementary file 1 [file DataSheet_1.zip › net_for_leaf/data/mydata11/train/image1/1/1050.png]

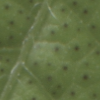

Supplement: Supplementary file 1 [file DataSheet_1.zip › net_for_leaf/data/mydata11/train/image1/1/1051.png]

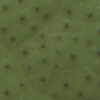

Supplement: Supplementary file 1 [file DataSheet_1.zip › net_for_leaf/data/mydata11/train/image1/1/1052.png]

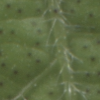

Supplement: Supplementary file 1 [file DataSheet_1.zip › net_for_leaf/data/mydata11/train/image1/1/1053.png]

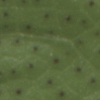

Supplement: Supplementary file 1 [file DataSheet_1.zip › net_for_leaf/data/mydata11/train/image1/1/1054.png]

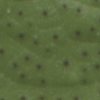

Supplement: Supplementary file 1 [file DataSheet_1.zip › net_for_leaf/data/mydata11/train/image1/1/1055.png]

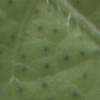

Supplement: Supplementary file 1 [file DataSheet_1.zip › net_for_leaf/data/mydata11/train/image1/1/1056.png]

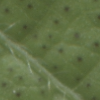

Supplement: Supplementary file 1 [file DataSheet_1.zip › net_for_leaf/data/mydata11/train/image1/1/1057.png]

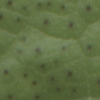

Supplement: Supplementary file 1 [file DataSheet_1.zip › net_for_leaf/data/mydata11/train/image1/1/1058.png]

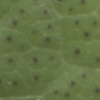

Supplement: Supplementary file 1 [file DataSheet_1.zip › net_for_leaf/data/mydata11/train/image1/1/1059.png]

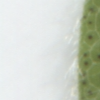

Supplement: Supplementary file 1 [file DataSheet_1.zip › net_for_leaf/data/mydata11/train/image1/1/106.png]

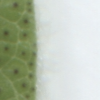

Supplement: Supplementary file 1 [file DataSheet_1.zip › net_for_leaf/data/mydata11/train/image1/1/1060.png]

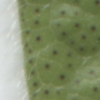

Supplement: Supplementary file 1 [file DataSheet_1.zip › net_for_leaf/data/mydata11/train/image1/1/1064.png]

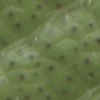

Supplement: Supplementary file 1 [file DataSheet_1.zip › net_for_leaf/data/mydata11/train/image1/1/1065.png]
